# Supplementary material for: Student Pharmacists during the Pandemic: Development of a COVID-19 Knowledge, Attitudes, and Practices (COVKAP) Survey
Source: Pharmacy (Basel). 2021 Sep 30;9(4):159. doi: 10.3390/pharmacy9040159 (PMC8544658; doi:10.3390/pharmacy9040159)
Supplement: Supplementary file 1 [file pharmacy-09-00159-s001.zip › pharmacy-1362360-supplementary.pdf]

Pharmacy students' knowledge, attitudes, and practices in COVID-19

Section I: Demographics

1. Age: ______ years
2. Sex: ☐M ☐F ☐Trans*
3. Are you of Hispanic or Latino origin? ☐Y ☐N
4. How would you describe yourself? ☐White ☐Black ☐Asian
   ☐Native Hawaiian or Pacific Islander ☐American Indian or Alaska Native
5. Graduation year: ___________
6. Status in Fall 2020: ☐Commute to campus ☐Live on campus ☐Online instruction only
   a. If living or commuting to campus, please provide the mode of transportation:

   ☐Drive self ☐Carpool ☐Shared transportation (such as University van)
   ☐Public transportation
7. Suppose that you need to return to school today. Whose responsibility is it to make these kinds of decisions?
   1)Solely mine
   2)Mostly mine
   3)Mine and my family
   4)Mostly the school
   5)Solely the school
8. Are you currently working as a pharmacy intern? ☐Y ☐N
9. Did you work in any pharmacy setting prior to pharmacy school? ☐Y ☐N
10. How many people including yourself are living in your household? _______
11. How many adults over 65 years living in your household? ______
12. Are you considered to be at higher risk in COVID-19 (as defined by the CDC as follows: People of any age with certain underlying medical conditions (CKD, COPD, Immunocompromised state, Obesity, Serious heart conditions, Sickle cell disease; Type 2 DM)?
    ☐Y ☐N
13. Do you have any family members considered to be at higher risk in COVID-19 (as defined by the CDC as follows: People of any age with certain underlying medical conditions (CKD, COPD, Immunocompromised state, Obesity, Serious heart conditions, Sickle cell disease; Type 2 DM)?
    ☐Y ☐N

Section II: Knowledge

Strongly disagree **Disagree** **Neutral** **Agree** **Strongly Agree**

1. The SARS-CoV-2 novel coronavirus was discussed in class during Spring 2020
2. COVID-19 is the disease caused by SARS-COV-2
3. Hydroxychloroquine is effective in treating COVID-19 patients
4. Remdesivir is effective in treating COVID-19 patients
5. COVID-19 develops only among the elderly
6. Eating or being in contact with wild animals would result in SARS-CoV-2 infections in humans
7. COVID-19 spreads through respiratory droplets of infected individuals
8. COVID-19 can spread asymptomatically

9. Use of personal protective equipment (e.g. masks) can protect individuals from getting infected
10. To prevent COVID-19 infection, individuals should avoid crowded places
11. If an individual travels to another state or region where COVID-19 cases are high, they should self-isolate upon their return

12. Clinical symptoms for COVID-19 include which of the following: (select all appropriate options)
    a. Fever
    b. Cough
    c. Fatigue
    d. Myalgia
    e. Loss of taste

Pharmacy students' knowledge, attitudes, and practices in COVID-19

Section II: Attitudes

Strongly disagree **Disagree** **Neutral** **Agree** **Strongly Agree**

1. I believe that COVID-19 is under control in the United States
2. I believe that adequate PPEs are available for all healthcare workers
3. I believe that adequate PPEs are available for members of the general public
4. I believe that information about COVID-19 testing and prevention should be available at all pharmacies.
5. I believe that pharmacists have adequate PPEs to protect themselves
6. I believe that pharmacies have adequate protections to prevent infections
7. I believe that pharmacists should participate in COVID-19 immunizations
8. I believe that pharmacists should participate in public health taskforces
9. I believe that it is important for me to counsel my family and friends on COVID-19
10. I believe that it is important for pharmacists to counsel the general public on COVID-19
11. I believe that patients coming to ask about COVID-19 related questions should ask the physician and not the pharmacist.
12. I believe that when a patient picks up a prescription and does not wear a mask, it has a negative impact on my interaction with him/her.
13. I believe that when a patient picks up a prescription and wears a mask, it has appositive impact on my interaction with him/her.
14. I believe that I should get vaccinated when the COVID-19 vaccine becomes available.
15. I believe that I am adequately prepared to administer the COVID-19 vaccine to patients

Section III: Practices

Never **1 time** **2-3 times** **Almost Every day** **Every day**

1. In the past 7 days, how often have you:
   a. Worn a mask in public?
   b. Visited restaurants or public services?
   c. Practiced social distancing?

2. In the past month, how often have you:
   a. Worn a mask in public?
   b. Visited restaurants or public services?
   c. Practiced social distancing?

3. When I have questions about COVID-19, I usually go to medical related journals to prepare myself.
4. When I have questions about COVID-19, I usually avoid social media sources.
5. I routinely conduct COVID-19 testing at the drive-through pharmacy.

Section IV: Returning to school in Fall 2020

1. Please rank your preference for resuming classes in Fall 2020:
   a. In class instruction with appropriate social distancing
   b. In class instruction with no social distancing
   c. Hybrid in-class and online instruction
   d. Online instruction only

Pharmacy students' knowledge, attitudes, and practices in COVID-19

2. Please list any health concerns that you may have in returning to school:

3. Please list any other concerns that you may have about returning to school:

4. Please list any concerns that you may have in returning to school:

5. Please provide 2-3 specific needs that your University needs to consider when resuming instruction in Fall 2020.
